# Effectiveness of Cognitive Behavioural Therapy on Sleep Outcome for Patients With Chronic Fatigue Syndrome in a Routine Clinical Service

**DOI:** 10.5964/ejop.16669

**Published:** 2026-08-28

**Authors:** Chotiman Chinvararak, Olivia Sterry, Trudie Chalder

**Affiliations:** 1 Department of Psychological Medicine, Division of Academic Psychiatry, King's College, London, United Kingdom; 2 Department of Psychiatry, Faculty of Medicine Vajira Hospital, Navamindradhiraj University, Bangkok, Thailand; 3 Persistent Physical Symptoms Research and Treatment Unit, London, United Kingdom; Stanford University, Stanford, USA

**Keywords:** chronic fatigue syndrome/myalgic encephalitis, cognitive behavioural therapy, sleep quality, cohort study

## Abstract

**Objective:**

We aimed to study the effectiveness of CBT for sleep difficulties in patients with chronic fatigue syndrome/myalgic encephalitis (CFS/ME).

**Method:**

We conducted a retrospective cohort study at the Persistent Physical Symptoms Research and Treatment Unit, Maudsley Hospital, between 2014 and 2019. The primary outcome was sleep quality and other sleep parameters assessed by the Pittsburgh Sleep Quality Index (PSQI). The secondary assessments comprised the Chalder Fatigue Questionnaire (CFS), the Work and Social Adjustment Scale (WSAS), and the Short-Form Health Survey (SF-36). The data were gathered at baseline, discharge, and follow-up periods (3, 6, and 12 months). A paired samples t-test, repeated measures ANOVAs, and the Friedman Test were used to determine the effectiveness of CBT. A linear mixed model was employed to evaluate the effect size of CBT.

**Results:**

From all 217 participants (71.4% female; mean age = 39.42 ± 12.32 years), 85.7% experienced poor sleep quality. The associated factors of poor sleep quality at baseline included primary education/secondary education/vocational qualification (OR_adj_ = 3.74, 95% CI [1.03, 13.64], *p* = .046), CFS score (OR_adj_ = 1.08, 95% CI [1.02, 1.15], *p* = .015), SF-36 score (OR_adj_ = 0.98, 95% CI [0.96, 0.99], *p* = .015), WSAS score (OR_adj_ = 1.06, 95% CI [1.02, 1.11], *p* = .004) and HADS-A score (OR_adj_ = 0.80, 95% CI [0.69, 0.93], *p* = .004). CBT had a substantial positive effect on sleep parameters, fatigue, and physical functioning over a 12-month follow-up period (*p* < .05).

**Conclusion:**

CBT had a significant positive effect on the sleep parameters. This effect persisted during a 12-month follow-up period. The results suggest CBT is an effective treatment for CFS/ME patients with sleep problems.

Chronic fatigue syndrome/myalgic encephalitis (CFS/ME) is a complicated multi-systemic disorder characterised by debilitating fatigue, post-exertional malaise (PEM), cognitive impairments, and sleep disturbance (Cairns & Hotopf, 2005). The age of onset is a bimodal distribution, ranging from 10 to 19 years and 30 to 39 years (Bakken et al., 2014; Rowe et al., 2017). Generally, the average age of onset is 33 (Bateman et al., 2021), and the onset of CFS/ME can be either sudden or gradual (Komaroff, 2015). The natural progression of illness is likely to be chronic and relapsing, with symptoms lasting at least 6 months (Komaroff, 2015). Furthermore, a systematic review study by Cairns and Hotopf in 2005 suggested that the complete recovery rate of CFS/ME patients is approximately just 5 per cent (Cairns & Hotopf, 2005).

There is no definitive biomarker or biological criteria to diagnose CFS/ME. Therefore, physicians must use the diagnostic criteria based on clinical symptoms and exclude other medical conditions associated with fatigue symptoms (Bateman et al., 2021). Examples of widely used diagnostic criteria are the Centers for Disease Control and Prevention (CDC) 2005 (CDC) empirical definition for CFS/ME by Reeves (Reeves et al., 2005), the Institute of Medicine (IOM) diagnostic criteria for systemic exertion intolerance disease (SEID) (Committee on the Diagnostic Criteria for Myalgic Encephalomyelitis/Chronic Fatigue Syndrome, 2015), and the NICE guideline for CFS/ME (National Collaborating Centre for Primary Care (UK), 2021). All criteria for CFS/ME descriptions are syndromal definitions with a set of major and minor criteria. The major criteria include debilitating fatigue, post-exertional malaise, and unrefreshing sleep or sleep disturbance.

The meta-analysis study by Lim et al. in 2020 revealed that the pooled prevalence of CFS/ME globally was around 0.89 per cent (Lim et al., 2020). The rate of CFS/ME in women tended to be greater than in men, approximately 1.5 to 2 times. Meanwhile, the NICE suggested the population prevalence in the UK is at least 0.2–0.4 per cent. These variations might result from differences in diagnostic criteria, case definition, and participants' recruitment procedures (Bayliss et al., 2016). 

The precipitating cause of CFS/ME cannot always be identified. For some, infectious illnesses such as the Epstein-Barr virus (EBV) are responsible. Other potential risk factors of CFS/ME are recurrent common cold infections, black or minority ethnic, being single, lower socioeconomic status, and family history of anxiety disorder (Lacerda et al., 2019). For most people, a combination of factors coming together likely trigger CFS/ME. However, for many, an apparent precipitant cannot be identified (Committee on the Diagnostic Criteria for Myalgic Encephalomyelitis/Chronic Fatigue Syndrome, 2015). 

Around 75 per cent of CFS/ME patients with moderate severity cannot return to work, and up to 25 per cent of patients with severe to very severe severity are housebound or bedridden (Pendergrast et al., 2016). Consequently, CFS/ME contributes to a significant burden on patients, families, and societies. Moreover, this illness substantially influences the nation's gross income. It is estimated that the total cost of care for patients with CFS/ME in the UK was projected to be about £3.3 billion in 2014/2015, with an average cost of £16,966 per person/year (Bayliss et al., 2016).

Sleep problems are one of the most common symptoms of CFS/ME. As a result, they are also a key item in the primary diagnostic criteria (Committee on the Diagnostic Criteria for Myalgic Encephalomyelitis/Chronic Fatigue Syndrome, 2015; National Collaborating Centre for Primary Care (UK), 2021). CFS/ME patients may experience a variety of sleep issues. Common manifestations are difficulties initiating insomnia, difficulty maintaining sleep, waking up earlier than desired, unrefreshing sleep, and hypersomnia (excessive sleepiness) (Jackson & Bruck, 2012). Therefore, physicians need to use subjective and objective methods to evaluate sleep problems depending on the suspected diagnosis. Objective evaluations comprise actigraphy and polysomnography (PSG) (Ibáñez et al., 2018). Subjective evaluations include a sleep diary and standardised self-report questionnaires. While the subjective methods are inexpensive and less time-consuming, PSG remains necessary to diagnose certain sleep disorders such as sleep-related breathing disorders, narcolepsy, or sleep-related behaviours (parasomnia) (Kushida et al., 2005). In addition, the multiple sleep latency test (MSLT) and the maintenance of wakefulness test (MWT) measure different aspects of daytime sleepiness and alertness (Kushida et al., 2005). Among various sleep questionnaires, the Pittsburgh Sleep Quality Index (PSQI) is acknowledged to be the most employed index of sleep quality (Fabbri et al., 2021). Early studies have demonstrated that the psychometric properties of PSQI have high internal consistency as well as discriminative validity in healthy populations and several medical settings (Mariman et al., 2013; Carpenter & Andrykowski, 1998; Buysse et al., 2008). However, Mariman et al. (2012) revealed that global PSQI might not be suitable to assess sleep quality in patients with CFS/ME since they found a low Cronbach's Alpha (0.64).

Several cross-sectional studies using PSQI have found that patients with CFS/ME had poor subjective sleep quality (Mariman et al., 2013; Maksoud et al., 2021; Josev et al., 2017; Neu et al., 2007). The multisite cohort study by Unger et al. (2017) indicated that approximately 90 per cent of CFS/ME patients reported unrefreshing or non-restorative sleep through questionnaires. Neu et al. (2007) also found that CFS/ME patients had prolonged sleep latency, decreased sleep efficiency, higher sleep disturbance, and daytime dysfunction compared to healthy controls. Furthermore, poor sleep quality was positively correlated with fatigue severity.

Interestingly, several PSG studies found no significant difference in sleep efficiency and sleep stages distribution between CFS/ME patients and normal controls (Jackson & Bruck, 2012; Maksoud et al., 2021; Josev et al., 2017; Neu et al., 2007; Russell et al., 2016). Furthermore, daytime sleepiness measured by the MSLT did not correlate with fatigue symptoms (Ibáñez et al., 2018). In addition, sleep quality determined by actigraphy could not anticipate next-day fatigue, while subjective sleep quality measured by sleep diaries could (Russell et al., 2016). One possible explanation for inconsistent data is that intraindividual day-to-day variability cannot be ascertained by a diagnostic overnight PSG (Fabbri et al., 2021). Tobback et al. (2015) demonstrated that significant differences in several objective sleep parameters between CFS patients and reference groups included a decrease in total sleep time and sleep efficiency, but an increase in sleep latency and wake after sleep onset. This finding suggested that the insomnia phenotype was present in CFS patients.

Thus, incompatibilities between subjectively poor sleep quality and objectively normal sleep architecture indicate that psychosocial or cognitive and behavioural factors might influence the perception of poor sleep quality in CFS/ME patients (Mariman et al., 2013). Poor sleep hygiene, excessive worry, preoccupation with symptoms, and psychiatric comorbidities could be potential explanations for sleep disturbance in CFS/ME patients.

Currently, there is no specific medical treatment for CFS/ME (Bateman et al., 2021) and no medication has been approved by the Medicines and Healthcare Products Regulatory Agency (MHRA) to treat this illness (White et al., 2011). Therefore, non-pharmacological and pharmacological therapy aims to relieve the symptoms and improve the patient's quality of life (Bateman et al., 2021; White et al., 2011). For non-pharmacological treatments, the Cochrane systematic review of 15 randomised controlled trials found that CBT effectively decreased fatigue scores at post-treatment compared with usual care (Adamson et al., 2020). Furthermore, there was no significant difference in the adverse events between the CBT and control groups.

Nevertheless, prior studies on non-pharmacological treatment for CFS/ME usually focus on fatigue and physical functioning as primary outcomes. There are still limited studies that assess the effectiveness of CBT on sleep quality in patients with CFS/ME (Russell et al., 2017). Therefore, this study aimed to evaluate whether CBT for CFS/ME patients improved sleep outcomes at discharge from treatment and follow-up at 3, 6, and 12 months in a routine clinical service. We used routinely collected data from the Persistent Physical Symptoms Research and Treatment Unit (PPS) at the Maudsley Hospital, previously a specialist service for CFS/ME in south London. We hypothesise that (1) There will be a high prevalence of poor sleep quality in patients with CFS/ME. (2) Poor sleep quality will be associated with fatigue, distress, and functioning in patients with CFS/ME. (3) There will be a low sleep efficiency because of a discrepancy between time in bed and total sleep time. (4) CBT will positively impact sleep by improving sleep quality and reducing fatigue.

## Method

### Study Design, Setting, and Participants

We conducted a retrospective cohort study (evaluation) using data collected routinely from the Persistent Physical Symptoms Research and Treatment Unit (PPS) at the Maudsley Hospital between 2014 and 2019. The service accepted referrals from general practitioners or hospital consultants. All participants were assessed by a medical practitioner during their ﬁrst appointment with the service.

### Inclusion/Exclusion Criteria

**Figure 1:**
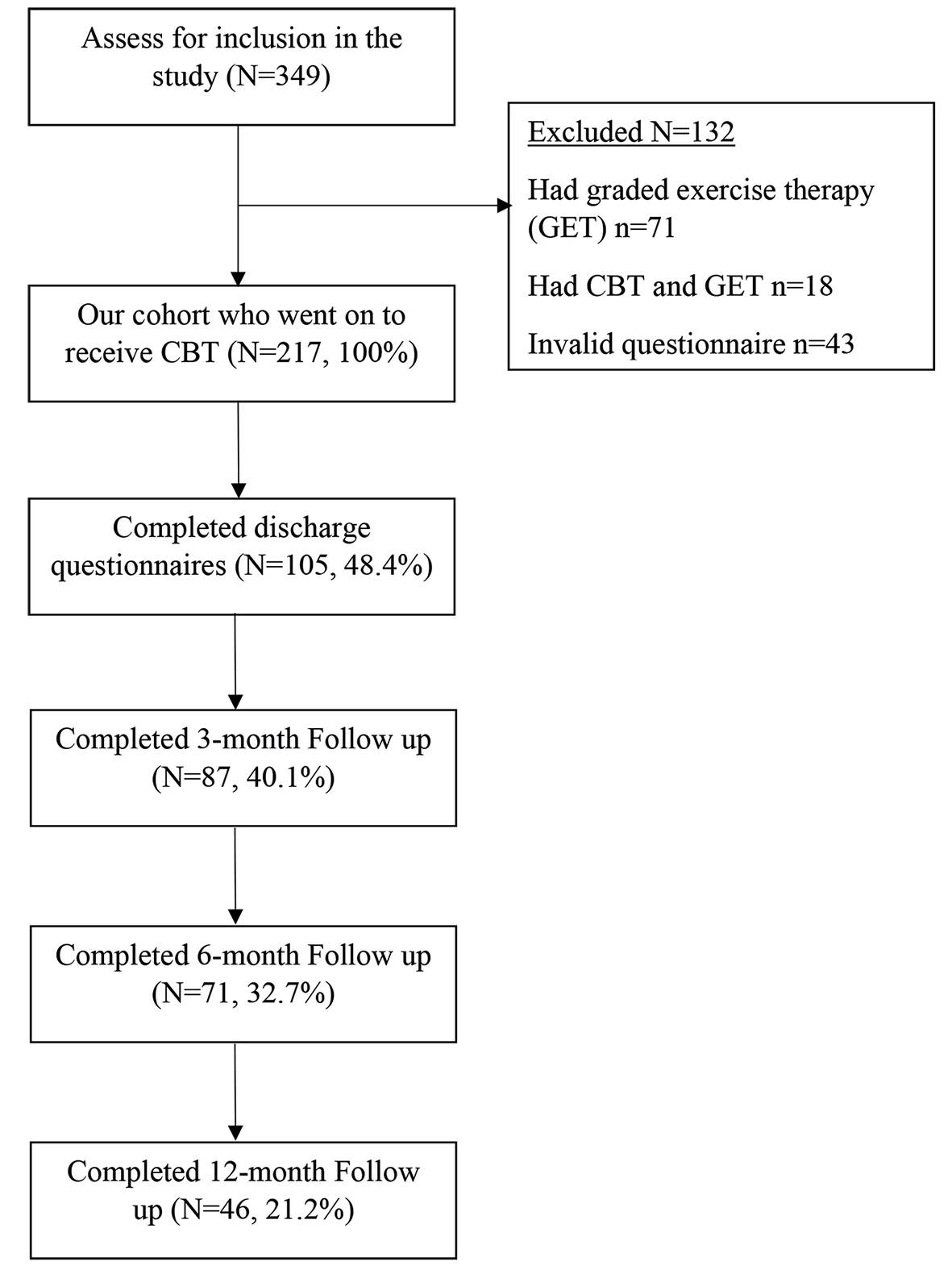
Flowchart Describing the Timeline of Patients, Including Exclusion Criteria

Participants were included if they: (a) were diagnosed with CFS/ME according to the NICE guidelines at that time [8]; (b) were aged 18 years and older; (c) had completed pre-treatment questionnaires; (d) received only CBT. Participants were excluded if they: (a) had active severe mental disorders such as schizophrenia, bipolar disorder, dementia, and intellectual disability; (b) had a medical condition that contributed to fatigue, such as a cardiac problem, cancer, acute infection, or metabolic disorders such as hypothyroidism; (c) had fatigue in the context of substance use disorder or medication use; (d) received other therapy such as graded exercise therapy (GET) or adaptive pacing therapy (APT) at the same time with CBT treatment. The flowchart of the study is illustrated in Figure 1.

### Ethics

Audit approval was provided by the Psychological Medicine Clinical Academic Group (ID number PPF191115) at the South London and Maudsley Hospital. All participants were asked to provide written consent to complete routine patient-reported outcome measures (PROMs). Informed consent, which described the purpose of data collection, i.e., to assess treatment effectiveness in the routine clinic, was obtained at the start of data collection.

### Cognitive Behavioural Therapy

Cognitive Behavioural Therapy (CBT; Adamson et al., 2020; Beck, 1997) is a structured, brief, and time-limited therapy. The number of individual sessions varied due to the individual's symptoms, treatment progression, and goals, but usually, patients were offered up to 16 sessions. The average duration was around 60 minutes. The objective of CBT in this context was to address the cognitive and behavioural factors interacting with the patient's physical symptoms, emotional distress, and disabilities. The first session consisted of an assessment in which therapists and participants collaboratively developed a case formulation, including identification and agreeing individual goals.

Therapeutic approaches included 1) Guiding patients to make links between symptoms, distress and cognitive behavioural coping responses, 2) Establishing a consistent pattern of rest and activity before collaboratively building up activity if required, 3) Addressing sleep difficulties via sleep hygiene, education, bed restriction and stimulus control 4) Problem-solving to help patients address emotional and social stress and increase a sense of bodily control.

CBT was provided by clinical psychologists or cognitive behaviour therapists who were specifically trained in CBT for CFS/ME.

### Measures

Participants were asked to complete self-reported questionnaires at the start of treatment, discharge, 3-month, 6-month, and 12-month follow-up. The questionnaires consisted of the following:

#### Participant Characteristics

Demographic data included age, sex, ethnicity, marital status, educational level, employment status, working hours, duration with CFS/ME, and comorbidity.

#### Sleep Quality

Sleep quality was evaluated by the Pittsburgh Sleep Quality Index (PSQI), a self-rated questionnaire developed by Buysse et al. (1989). It consists of 19 individual items that generate 7 component scores, including 1) subjective sleep quality, 2) sleep latency, 3) sleep duration, 4) sleep efficiency, 5) sleep disturbance, 6) use of sleep medication, and 7) daytime dysfunction. Each item is rated from 0 to 3, giving a global PSQI score between 0 and 21. A global score greater than 5 indicates poor sleep quality. At this cut-off, PSQI yields a diagnostic sensitivity of 89.6 per cent and specificity of 86.5 per cent in distinguishing good and poor sleepers (Buysse et al., 1989).

Sleep latency (SL) or sleep onset latency (SOL) is the time it takes a participant to fall asleep after turning the lights off. In general, healthy people usually take between 10 and 20 minutes to fall asleep. Sleep duration or total sleep time (TST) is defined as the total amount of sleep that a participant obtains per day. Sleep efficiency (SE) is the percentage calculated by TST divided by total time in bed (TIB). Sleep disturbance consists of the items assessing sleep environment, sleep-related breathing problems, nocturia, nightmares, and other symptoms reported by a participant. Finally, the daytime function is assessed by the severity of daytime sleepiness and subjective feeling of enthusiasm to get things done during the day (Buysse et al., 1989). The internal consistency and CFA of the PSQI in this study are shown in the appendix. 

#### Fatigue

Fatigue was measured using the fatigue questionnaire (CFS) developed by Chalder et al. (1993). It comprises 11 self-reported items, with a Likert scale scoring 0 to 3. A total score ranges from 0 to 33, with the lowest score indicating the least fatigue. The CFS was validated in CFS/ME populations, and its Cronbach's Alpha was 0.88, reflecting good internal consistency. Moreover, the area under the curve for ROC was 0.91, showing good discrimination of CFS/ME from normal subjects (Cella & Chalder, 2010).

#### Work and Social Adjustment

The Work and Social Adjustment Scale (WSAS) was used to assess participation in activities and daily life. It has five self-rated items using a 0 to 8 scale. This questionnaire was first introduced by Marks in 1986 (Marks, 1986). A total WSAS score ranges from 0 to 40, with a higher score representing a poorer social adjustment or a higher level of disability. In contrast, lower scores on the WSAS were modestly associated with better physical functioning. Moreover, it has been validated in CFS/ME patients with good reliability (Cella et al., 2011).

#### Physical Functioning

Physical functioning was assessed by the physical functioning subscale (PF-10) of the Short-Form Health Survey (SF-36). It contains 10 self-reported items and a total score ranging from 0 to 100. A higher score indicates better physical functioning. PF-10 has been validated in patients with CFS/ME and has good internal consistency with Cronbach's Alpha = 0.90. The items of PF-10 represent a unidimensional construct of physical functioning for most patients, and the item structure is reproducible across divergent patient samples (Ware & Sherbourne, 1992; Buchwald et al., 1996; Haley et al., 1994).

#### Anxiety and Depression

The Hospital Anxiety and Depression Scale (HADS) is a self-rating scale developed by Zigmond et al. in 1983 to assess psychological distress in medical outpatient clinic settings. It includes 14 items: seven for the anxiety subscale (HADS Anxiety) and seven for the depression subscale (HADS Depression). The total score of each dimension is 21, and a score greater than 10 on each subscale indicates anxiety or depression. This cut-off value has a sensitivity of 75.7 per cent and a specificity of 66.3 per cent for screening depression, 65.7 per cent, and 71.3 per cent for screening anxiety (Zigmond & Snaith, 1983; Mitchell et al., 2010).

### Statistical Analyses

Statistical analyses were conducted using SPSS Version 28 (SPSS Inc., Chicago, IL) and STATA Version 16 (STATA Corp, US). Descriptive statistics were used to analyse demographic data, the sleep parameters, and the prevalence of poor sleep quality (including frequency, percentage, mean, median, minimum, maximum, standard deviation, and interquartile range).

Multivariable regression analyses were used to determine factors associated with poor sleep quality adjusted for age and sex.

Sleep duration (hours) at baseline was compared to the average sleep duration in the normal adult population (7 hours) using the one-sample t-test. Furthermore, independent t-test, chi-square, and Mann–Whitney U test were used to compare the characteristics between the follow-up and non-follow-up groups. Participants who completed measures at least once during the discharge period were categorised as the follow-up group, while the others were identified as the non-follow-up group.

The effectiveness of CBT on sleep efficiency, sleep duration, and PSQI scores at the discharge and the follow-up period was assessed by a paired samples *t*-test and repeated measures ANOVAs. The Friedman Test was applied for sleep latency, as the data showed a skewness. A linear mixed model was used to estimate the effect size of CBT on the sleep parameters and secondary outcomes; we used the listwise deletion method for handling missing data. For all tests, a *p*-value < .05 indicated statistical significance.

We assessed the internal consistency of the PSQI by calculating Cronbach's alpha for the global and subscales of the PSQI in our participants. We used Pearson's correlation to evaluate the correlation between each item of the PSQI. Finally, we performed confirmatory factor analysis (CFA) to confirm that the structural model from our data was compatible with the pre-defined theoretical model.

## Results

### Participants

Figure 1 demonstrates the flow of participants throughout this study. Of the 349 patients who entered the PPS service for assessment, 217 patients received CBT and met the selection criteria.

One hundred and fifty-five (71.4%) participants were female, and 62 (28.6%) were male. The participants were between 18 and 72 years (mean age 39.42 years, *SD* = 12.33); 177 (81.6%) were white. Ninety-eight participants (45.2%) were single, and 95 (43.8%) were married or living together. Furthermore, 73 (33.6%) participants obtained an undergraduate degree, and 57 (26.3%) were university postgraduate. Among 119 (54.8) who were employed, the median working hours was 30 (IQR 16-40). In addition, participants had been diagnosed with CFS/ME for a median duration of 3.8 years (IQR 1.4-8.5); 29 (13.4%) had at least one comorbidity. Finally, the median CBT treatment sessions was 13 (IQR 10-16) (see Table 1).

**Table 1:** Participant's Characteristics at Baseline (n = 217)

Characteristics	*N* (%) or Mean±*SD*
Age (years) min = 18 max = 72	39.42 ± 12.33
Sex	
Female	155 (71.4)
Male	62 (28.6)
Ethnic group	
White	177 (81.6)
Black/African/Caribbean/Black British	17 (7.8)
Mixed/ Multiple ethnic groups	9 (4.1)
Asian or Asian British	6 (2.8)
Other ethnic groups	3 (1.4)
Unknown	5 (2.3)
Marital status	
Single	98 (45.2)
Married/Living together	95 (43.8)
Divorced/Separated/Widowed	19 (8.8)
Unknown	5 (2.3)
Educational qualification	
None	3 (1.4)
Primary education	15 (6.9)
Secondary school to A levels	27 (12.4)
Vocational qualification	31 (14.3)
University undergraduate	73 (33.6)
University postgraduate	57 (26.3)
Other	7 (3.2)
Unknown	4 (1.8)
Employment	
No	73 (33.6)
Yes	119 (54.8)
Unknown	25 (11.5)
Working hours; median (IQR)	30 (16 –40)
Duration with CFS/ME (years); median (IQR)	3.8 (1.4 –8.5)
Comorbidity	29 (13.4)
Fatigue (CFS)	26.02 ± 5.82
Physical functioning (SF-36)	45.21 ± 28.66
Work and social adjustment (WSAS)	25.98 ± 9.26
Anxiety (HADS-A)	9.77 ± 2.71
Normal	131 (60.4)
Anxiety	86 (39.6)
Depression (HADS-D)	9.11 ± 2.09
Normal	166 (76.5)
Depression	51 (23.5)
Number of active treatment sessions; median (IQR)	13 (10–16)

*Note*. Data are presented as number (%), mean ± standard deviation or median (interquartile range).

### Prevalence of Poor Sleep Quality

Table 2 reports the sleep parameters of participants at baseline. Within our cohort, 186 participants (85.7%) had a score greater than 5 on the PSQI, indicating the presence of poor sleep quality. The means of PSQI, sleep duration, and sleep efficiency were 9.93 (*SD* = 4.18) points, 7.01 (*SD* = 1.79) hours, and 77.98 (*SD* = 16.00) per cent, respectively. The median sleep latency was 30 (IQR 15-60) minutes.

**Table 2:** Sleep Parameters at Baseline (n = 217)

Sleep parameters	*N* (%) or Mean±*SD*
Sleep latency (minutes)	30 (15–60)
Sleep duration (hours)	7.01 ± 1.79
Sleep efficiency	77.98 ± 16.00
Total PSQI score	9.93 ± 4.18
Poor sleep quality (PSQI > 5)	186 (85.7)
Good sleep quality (PSQI ≤ 5)	31 (14.3)

*Note*. Data are presented as number (%), mean ± standard deviation or median (interquartile range).

### Comparison of Characteristics Between Those With and Without a Follow Up Measure

There was a significant difference in marital status (χ2 = 10.666, *df* = 3, *p* = .011), educational qualification (exact *p* = .026), working hours (Mann-Whitney U = 1,1176.5, *p* = .027), anxiety (*t* = 2.476, *df* = 214.974, *p* = .014) and depression (*t* = -3.226, *df* = 214.98, *p* < .001) between the follow-up and non-follow-up group. Compared to the follow-up group, the non-follow-up group had a higher proportion of divorced, separated, or widowed individuals, higher working hours and higher HADS-D scores. By contrast, the follow-up group had a higher proportion of university undergraduate or postgraduate degrees and a higher HADS-A score (see Table 3).

**Table 3:** Comparison of Characteristics Between Those With and Without a Follow Up Measure

Characteristics	Follow-up (*n* = 113)	Non-follow up (*n* = 104)	*p*
Age (years)	39.63 ± 12.12	39.18 ± 12.61	.792
Sex			
Female	76 (67.3)	79 (76.0)	.156
Male	37 (32.7)	25 (24.0)	
Ethnic group			
White	90 (79.6)	87 (83.7)	.670
Black/African/Caribbean/	11 (9.7)	6 (5.8)	
Black British			
Mixed/ Multiple ethnic groups	6 (5.3)	3 (2.9)	
Asian or Asian British	2 (1.8)	4 (3.8)	
Other ethnic groups	1 (0.9)	2 (1.9)	
Unknown	3 (2.7)	2 (1.9)	
Marital Status			
Single	56 (49.6)	42 (40.4)	.011*
Married/Living together	52 (46.0)	43 (41.3)	
Divorced/separated/widowed	4 (3.5)	15 (14.4)	
Unknown	1 (0.9)	4 (3.8)	
Highest educational qualification			
None	1 (0.9)	2 (1.9)	.026*
Primary education	9 (8.0)	6 (5.8)	
Secondary school to A levels	13 (11.5)	14 (13.5)	
Vocational qualification	15 (13.3)	16 (15.4)	
University undergraduate	41 (36.3)	32 (30.8)	
University postgraduate	34 (30.1)	23 (22.1)	
Other	0 (0.0)	7 (6.7)	
Unknown	0 (0.0)	4 (3.8)	
Employment			
No	34 (30.1)	39 (37.5)	.588
Yes	69 (61.1)	50 (48.1)	
Unknown	10 (8.8)	15 (14.4)	
Working hours	27.5 (15–37.5)	35.8 (24–42)	.027*
Comorbidity			
No	97 (85.8%)	91 (87.5%)	.578
Yes			
Depression	6 (5.3)	3 (2.9)	
Anxiety disorder	3 (2.7)	4 (3.8)	
Bipolar depression	0 (0.0)	1 (1.0)	
Somatoform disorder	6 (5.3)	2 (1.9)	
Reaction to stress	0 (0.0)	1 (1.0)	
Personality disorder	0 (0.0)	1 (1.0)	
Medical conditions	1 (0.9)	1 (1.0)	
Duration of CFS/ME (years)	3.5 (2–8)	4.8 (2–10)	.380
Fatigue (CFS)	25.48 ± 5.63	26.61 ± 5.98	.154
Physical functioning (SF-36)	46.81 ± 27.22	43.46 ± 30.17	.390
Work and social adjustment (WSAS)	25.45 ± 8.74	26.56 ± 9.81	.381
Anxiety (HADS-A)	10.20 ± 2.79	9.31 ± 2.54	.014*
Normal	60 (53.1)	71 (68.3)	
Anxiety	53 (46.9)	33 (31.7)	
Depression (HADS-D)	8.68 ± 2.14	9.58 ± 1.95	< .001**
Normal	98 (86.7)	68 (65.4)	
Depression	15 (13.3)	36 (34.6)	

*Note*. Data are presented as number (%), mean ± standard deviation or median (interquartile range).

*p < .05. **p < .01.

### Factors Associated With Poor Sleep Quality at Baseline

Table 4 shows the univariable and multivariable analyses of baseline associations between explanatory variables and poor sleep quality. When adjusting for age and sex, primary education/secondary education/vocational qualification (OR_adj_ = 3.74, 95% CI [1.03, 13.64], *p* = .046), fatigue score (OR_adj_ = 1.08, 95% CI [1.02, 1.15], *p* = .015), SF-36 score (OR_adj_ = 0.98, 95% CI [0.96, 0.99], *p* = .015), WSAS score (OR_adj_ = 1.06, 95% CI [1.02, 1.11], *p* = .004) and HADS-A score (OR_adj_ = 0.80, 95% CI [0.69, 0.93], *p* = .004) remained as independently associated with poor sleep quality at baseline. Higher HADS-A and better physical functioning scores were associated with a lower risk of poor sleep quality. In contrast, higher fatigue, higher WSAS score, and lower education level, i.e., not graduates, were associated with a higher risk of poor sleep quality.

**Table 4:** Factors Associated With Poor Sleep Quality Using Univariable and Multivariable Regression Analysis

Variable	Univariable analysis			Multivariable analysis		
	OR^a^	95% CI	*p*	OR_adj_^b^	95% CI	*p*
Ethnic group						
White	1.00	Reference		1.00	Reference	
Others	2.32	(0.67 - 8.04)	0.185	2.06	(0.55 - 7.73)	0.284
Marital status						
Single	1.00	Reference		1.00	Reference	
Married/Living together	1.06	(0.49 - 2.32)	0.876	1.22	(0.51 - 2.92)	0.655
Divorced/separated/widowed	3.31	(0.41- 26.58)	0.260	2.52	(0.28-22.95)	0.412
Highest educational qualification						
Primary education/Secondary	3.08	(0.97 - 9.71)	0.056	3.74	(1.03- 13.64)	0.046*
Education/Vocational qualification						
University undergraduate	0.62	(0.25 - 1.51)	0.291	0.72	(0.28 - 1.88)	0.506
University postgraduate	1.00	Reference		1.00	Reference	
Employment						
No	1.00	Reference		1.00	Reference	
Yes	0.54	(0.23 - 1.29)	0.168	0.74	(0.28 - 1.91)	0.532
Unknown	2.95	(0.35- 24.88)	0.319	2.81	(0.32-24.75)	0.352
Comorbidity						
No	1.00	Reference		1.00	Reference	
Yes	1.52	(0.43 - 5.35)	0.517	1.43	(0.40 - 5.08)	0.580
Duration of CFS/ME (years)	1.00	(0.94 - 1.07)	0.945	1.00	(0.93 - 1.07)	0.995
Fatigue (CFS)	1.08	(1.02 - 1.15)	0.012**	1.08	(1.02 - 1.15)	0.015**
Physical functioning (SF-36)	0.98	(0.97 - 0.99)	0.003**	0.98	(0.96 - 0.99)	0.004**
Work and social adjustment (WSAS)	1.07	(1.02 - 1.11)	0.003**	1.06	(1.02 - 1.11)	0.004**
Anxiety (HADS-A)	0.80	(0.69 - 0.93)	0.003**	0.80	(0.69 - 0.93)	0.004**
Depression (HADS-D)	1.01	(0.84 - 1.22)	0.894	1.01	(0.84 - 1.22)	0.897

*Note*. OR = Odds Ratio; OR_adj_ = Adjusted Odds Ratio; CI = confidence interval.

^a^Crude Odds Ratio is estimated by Binary Logistic regression. ^b^Adjusted Odds Ratio is estimated by Multiple Logistic regression adjusted by age and sex.

*p < .05. **p < .01.

### Comparison of Sleep Duration Between Participants and the Healthy Adult Population

In our cohort, the mean sleep duration was 7.01 (*SD* = 1.79) hours. The difference was not statistically significant from the average sleep duration in the healthy adult population (*t* = 0.066, *df* = 207, *p* = 0.947; see Table 5).

**Table 5:** Comparison of Sleep Duration Between Participants and Healthy Adult Population

Sleep duration (hours)				Mean difference	95% CI		*p*
Participants	Normal population	*t*	*df*		Lower	Upper	
7.01 ± 1.79	7.00	0.066	207	0.0082	-0.236	0.252	.947

### Changes in Sleep Parameters Over Time

**Figure 2:**
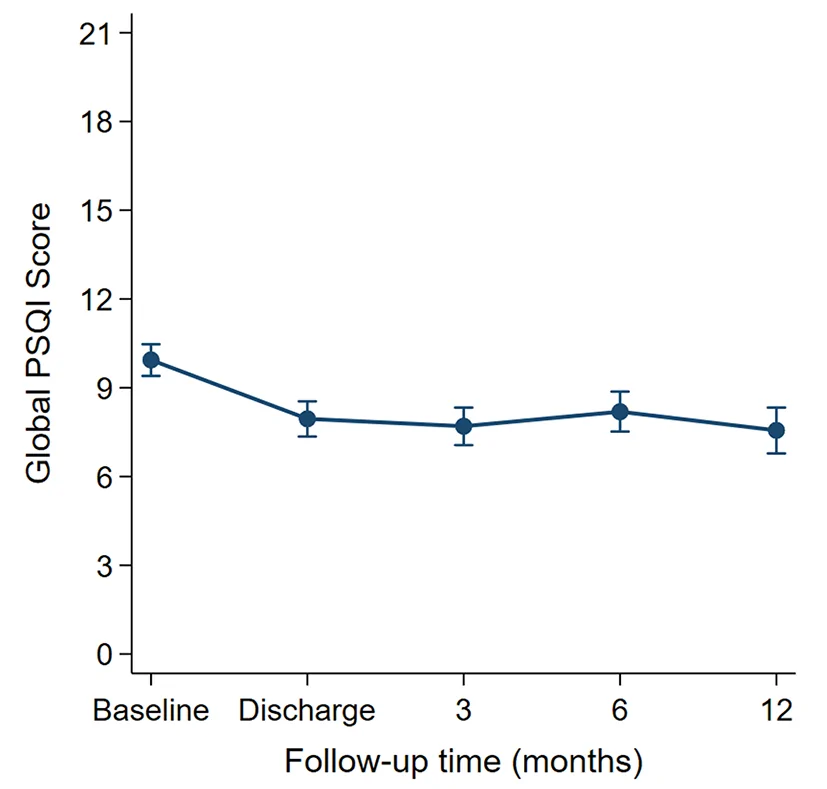
PSQI Scores Across Time Points *Note*. Error bars of standard error are included.

**Figure 3:**
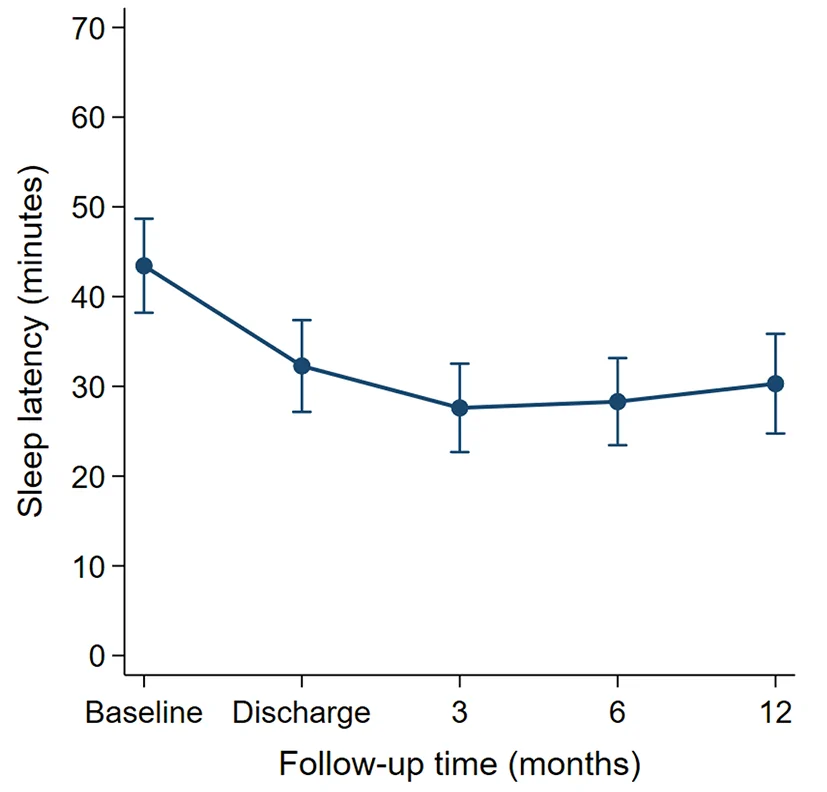
Sleep Latency Across Time Points *Note*. Error bars of standard error are included.

**Figure 4:**
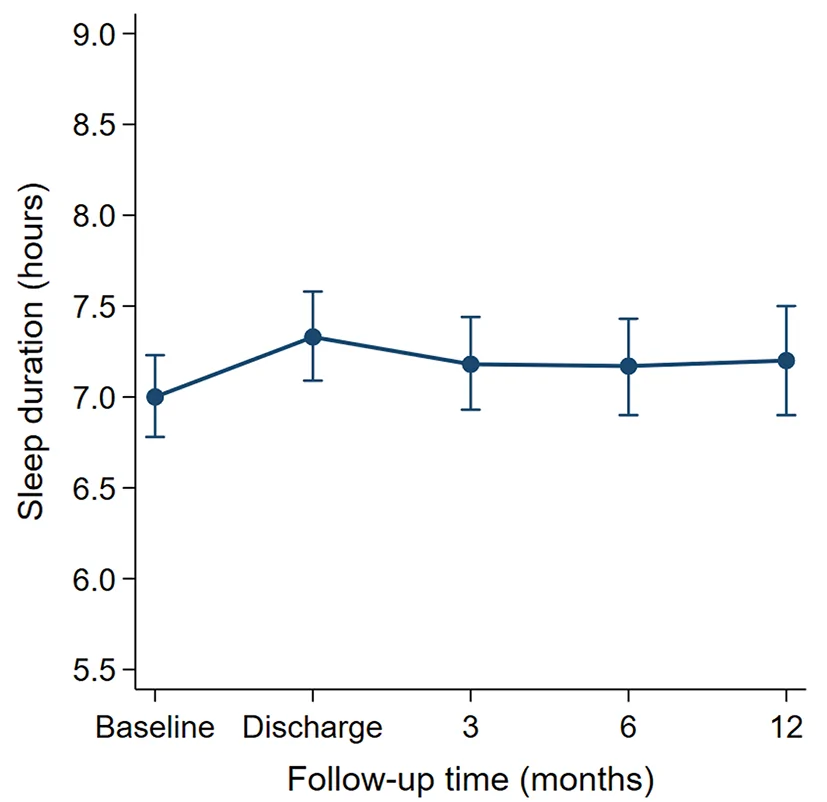
Sleep Duration Across Time Points *Note*. Error bars of standard error are included.

**Figure 5:**
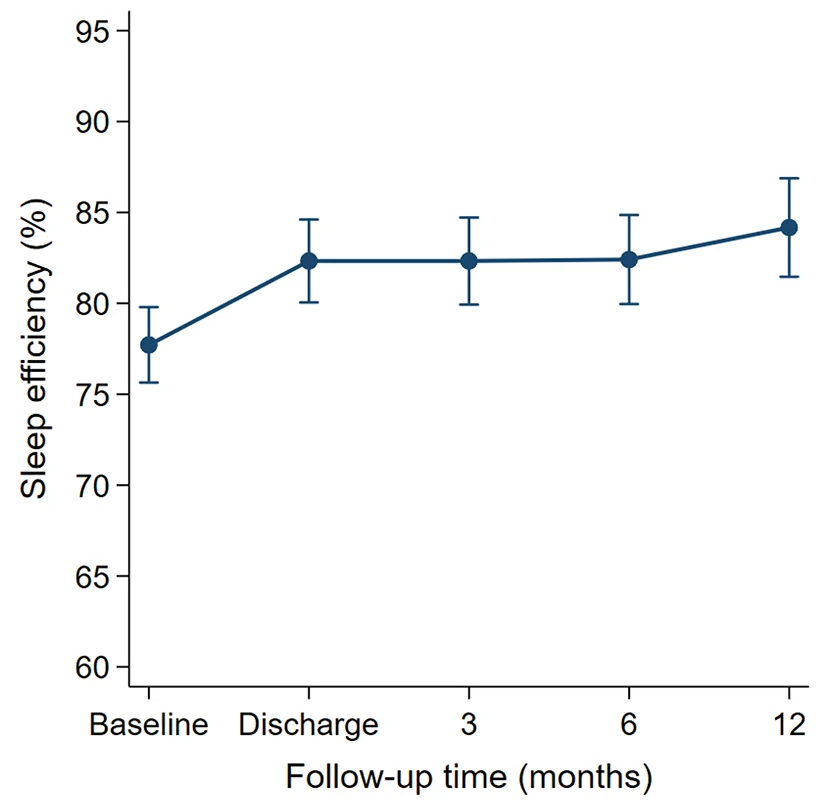
Sleep Efficiency Across Time Points *Note*. Error bars of standard error are included.

Estimated means and mean changes in sleep parameters, including PSQI score, sleep latency, sleep duration, and sleep efficiency, are shown in Table 6, Figures 2 to 5. When applying a linear mixed model to estimate the effect sizes, CBT had a significant effect over time on participants' PSQI global score, sleep latency, and sleep efficiency.

**Table 6:** Estimated Means and Mean Changes for Outcomes Over Time (Missing Values Handled by the Listwise Deletion Method)

Outcomes	*n*	Mean ± *SD*	Change from baseline (95% CI)		*p*
Sleep quality (PSQI)					
Global PSQI Score					
Baseline	217	9.93 ± 4.18		Reference	
Discharge	130	7.34 ± 3.42	-1.99	(-2.51, -1.47)	< .001*
3 months	99	7.09 ± 3.90	-2.24	(-2.82, -1.66)	< .001*
6 months	87	7.74 ± 4.02	-1.74	(-2.39, -1.10)	< .001*
12 months	66	6.61 ± 3.59	-2.38	(-3.14, -1.62)	< .001*
Sleep latency (minutes)					
Baseline	211	43.29 ± 44.05		Reference	
Discharge	128	30.63 ± 27.41	-11.18	(-15.90, -6.46)	< .001*
3 months	98	25.23 ± 23.70	-15.84	(-21.16, -10.53)	< .001*
6 months	87	27.99 ± 24.68	-15.15	(-21.17, -9.14)	< .001*
12 months	66	28.74 ± 27.34	-13.15	(-20.33, -5.96)	< .001*
Sleep duration (hours)					
Baseline	208	7.01 ± 1.79		Reference	
Discharge	127	7.42 ± 1.42	0.33	(0.13, 0.53)	.002*
3 months	97	7.07 ± 1.23	0.18	(-0.05, 0.40)	.125
6 months	86	7.15 ± 1.48	0.16	(-0.09, 0.41)	.213
12 months	66	7.33 ± 1.45	0.20	(-0.10, 0.50)	.188
Sleep efficiency (%)					
Baseline	205	77.98 ± 16.00		Reference	
Discharge	127	82.75 ± 12.72	4.62	(2.40, 6.84)	< .001*
3 months	96	82.39 ± 12.90	4.62	(2.18, 7.05)	< .001*
6 months	85	82.37 ± 13.39	4.70	(2.08, 7.31)	< .001*
12 months	66	85.12 ± 11.96	6.46	(3.51, 9.41)	< .001*
Fatigue (CFS)					
Baseline	220	26.08 ± 5.80		Reference	
Discharge	130	18.92 ± 7.91	-6.98	(-8.10, -5.85)	< .001*
3 months	102	18.97 ± 7.43	-6.62	(-7.86, -5.38)	< .001*
6 months	88	17.98 ± 7.33	-7.57	(-8.92, -6.22)	< .001*
12 months	67	17.21 ± 7.04	-8.27	(-9.81, -6.72)	< .001*
Work and social adjustment (WSAS)					
Baseline	221	26.14 ± 9.26		Reference	
Discharge	131	20.11 ± 10.11	-5.80	(-6.87, -4.73)	< .001*
3 months	102	18.50 ± 9.96	-6.91	(-8.19, -5.63)	< .001*
6 months	88	19.17 ± 10.70	-6.26	(-7.81, -4.70)	< .001*
12 months	67	17.21 ± 10.74	-6.79	(-8.73, -4.85)	< .001*
Physical functioning (SF-36)					
Baseline	221	45.25 ± 28.81		Reference	
Discharge	131	58.15 ± 28.07	12.18	(9.31, 15.05)	< .001*
3 months	101	63.17 ± 27.33	14.31	(10.91, 17.71)	< .001*
6 months	88	60.92 ± 29.63	12.99	(8.90, 17.08)	< .001*
12 months	66	66.76 ± 26.40	14.46	(9.33, 19.58)	< .001*

*Note*. Analyses were conducted with the use of a linear mixed-effects model with an unstructured correlation matrix.

*p < .01.

### Changes in Fatigue, WASA and SF-36 Over Time

Estimated means and mean changes in the secondary outcomes, including fatigue, SF-36, and WSAS, are shown in Table 6, Figures A2 to A4. When applying a linear mixed model to estimate the effect sizes, CBT had a significant effect over time on participants' fatigue, physical functioning and WSAS.

### Internal Consistency and Confirmatory Factor Analysis (CFA) of the PSQI in our CFS/ME Participants

Table A1 shows the internal consistency of the PSQI. We found that the Cronbach's alpha of PSQI was 0.731, which reflects good internal consistency. Pearson's correlation also showed that the items of the PSQI correlated with each other significantly (*p* < .05, see Table A2).

**Figure A1:**
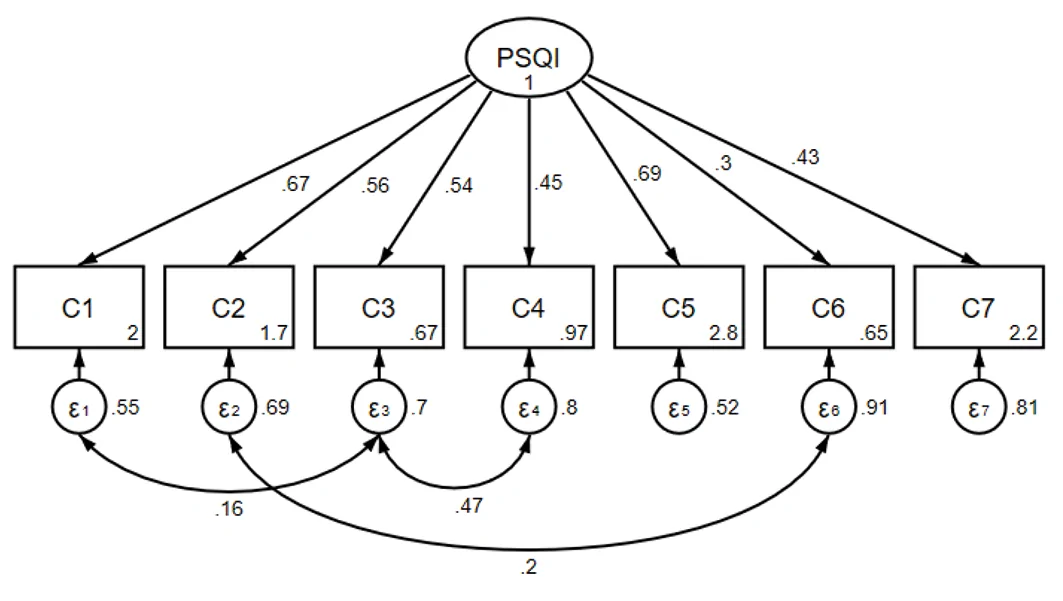
Confirmatory Factor Analysis (Adjusted Model With B)

**Figure A2:**
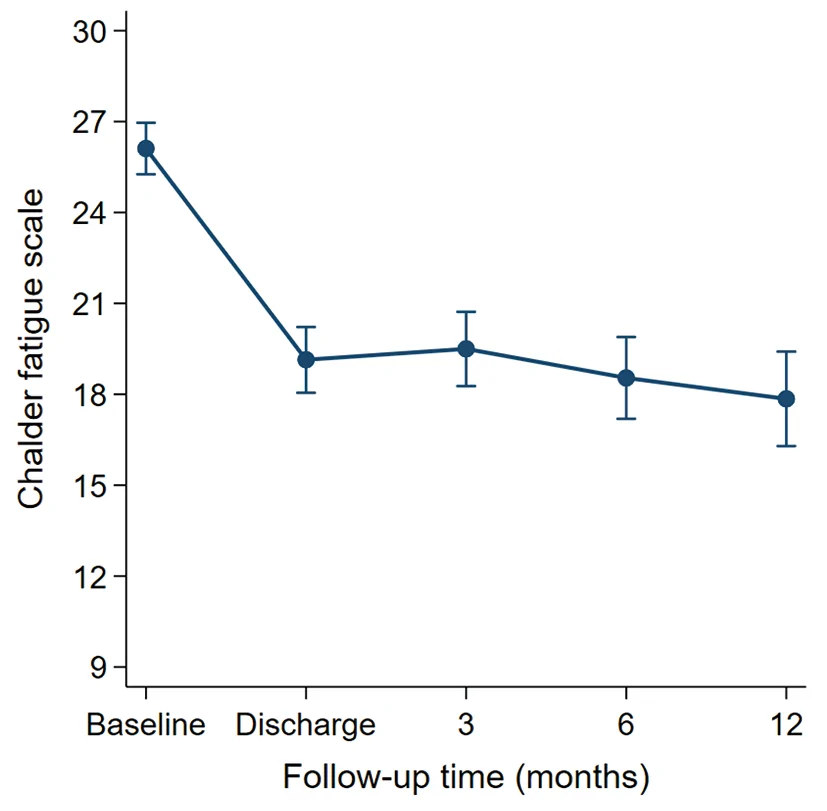
CFS Across Time Points *Note*. Error bars of standard error are included.

**Figure A3:**
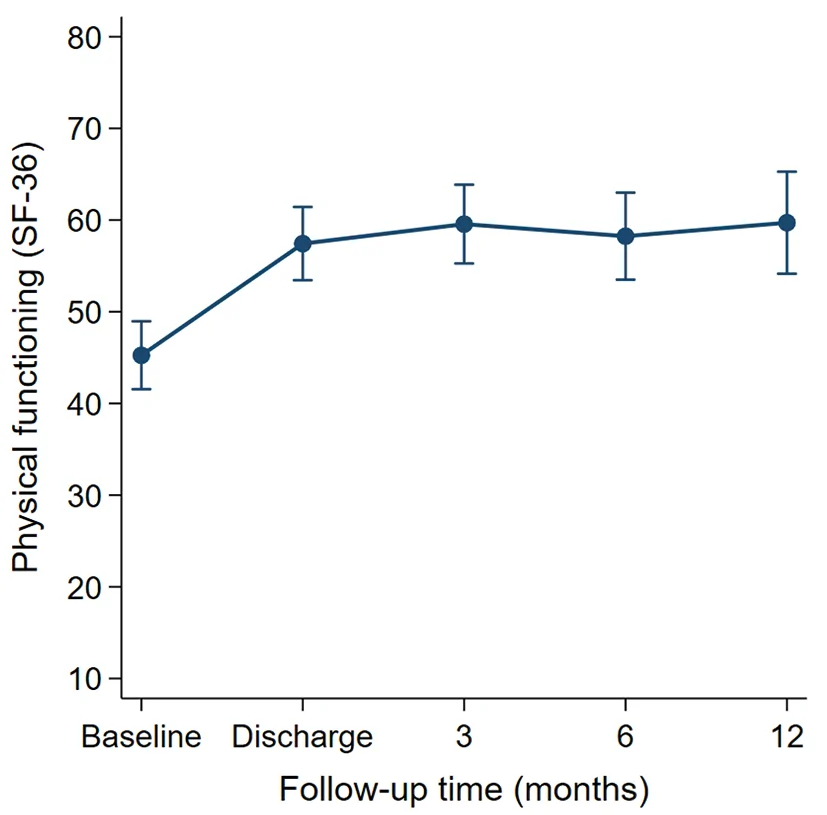
SF-36 Score Across Time Points *Note*. Error bars of standard error are included.

**Figure A4:**
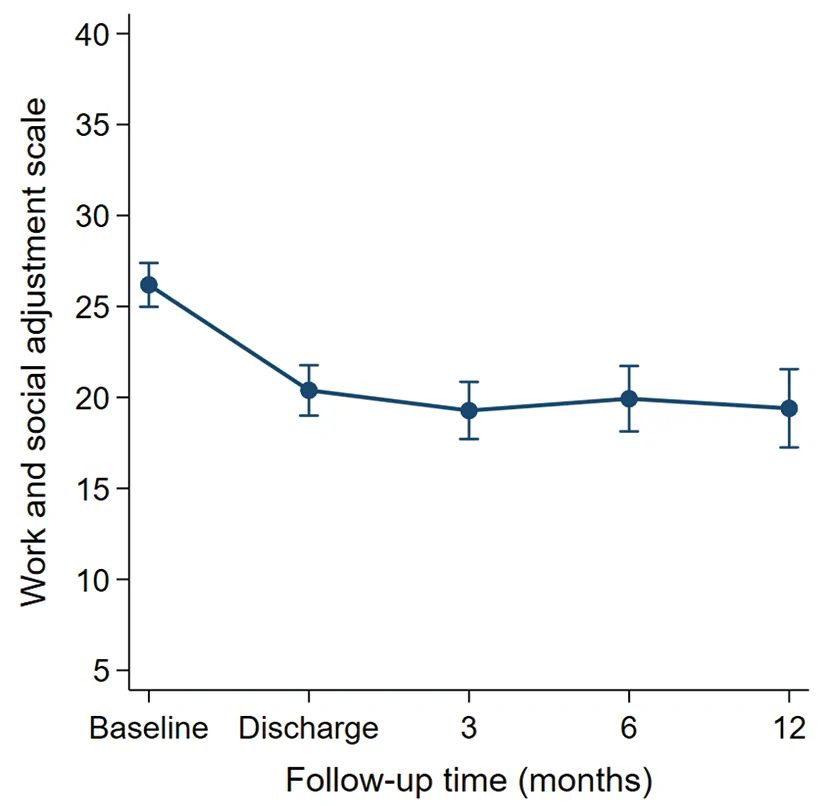
WSAS Across Time Points *Note*. Error bars of standard error are included.

The CFA demonstrates that the adjusted structural model of the PSQI was compatible with the PSQI's theory (see Table A3 and Table A4, Figure A1).

## Discussion

To the best of our knowledge, this study is the first retrospective cohort study (evaluation) to investigate CBT's effectiveness on sleep outcomes using a comprehensive measure in participants with CFS/ME in a real-world clinical service. At baseline (before treatment), there was a high prevalence of poor sleep quality in CFS/ME (85.7%). Furthermore, poor sleep quality was associated with lower educational levels and severity of fatigue, while better physical functioning was associated with better sleep quality.

The prevalence of poor sleep quality was similar to early cross-sectional studies, which demonstrated a high prevalence of poor sleep quality ranging between 56 and 100 per cent in CFS/ME patients (Jackson & Bruck, 2012; Mariman et al., 2013; Maksoud et al., 2021). Participants' characteristics, such as age group, comorbidity, ethnicity, and differences in the sleep measure, may have affected the variation in prevalence rates.

We found that sleep duration in our cohort did not significantly differ from the average sleep duration in the healthy adult population (7 hours) (Hirshkowitz et al., 2015). This finding likely suggests that CFS/ME patients may suffer from a sleep quality problem rather than sleep quantity. In this study, we used only PSQI to assess total sleep time, while Tobback et al. (2015) showed that there were notable differences in various objective sleep measures, such as total sleep time from PSG, between CFS/ME patients and control groups.

We did not find a correlation between depression and sleep quality. The study by Neu et al. (2007) also revealed that subjective poor sleep quality was not correlated with affective symptoms (Josev et al., 2017). Interestingly, we found that higher anxiety severity was associated with a lower risk of poor sleep quality. This finding contradicts prior studies, which demonstrated that higher anxiety levels are often associated with poorer subjective sleep quality (Castro-Marrero et al., 2018; Josev et al., 2017). Most participants in our study had mild anxiety since we excluded participants with active severe mental disorders. It is therefore possible that this accounts for why wedid not see the typical relationship between anxiety and sleep quality. Or anxiety symptoms in CFS/ME patients may impact sleep differently from anxiety symptoms in other mental health disorders.

We assessed outcomes across time after CBT and at follow-up to 12 months. Compared to the follow-up group, the non-follow-up group tended to have a lower education level, higher working hours, and higher depression. Interestingly, those who provided follow-up were more anxious than those who did not provide follow-up measures. Jin et al. (2008) found that demographic characteristics were not predictive of poorer treatment compliance. Their study showed that patients' beliefs, motivation, negative attitudes toward therapy, and therapy-related factors significantly affected treatment compliance (Jin et al., 2008). Moreover, Jin et al. suggested that participants with anxiety adhered to treatment, perhaps because they were more concerned with their physical symptoms and wanted to discuss their concerns with a therapist.

We found that the CBT intervention significantly improved participants' sleep quality, sleep latency, and sleep efficiency. The effect size of mean changes in PSQI score and sleep efficiency was modest. However, it is quite large for reducing sleep latency during follow-up periods. The improvement in sleep duration was significant at the end of therapy assessment, while the other follow-ups remained stable. Nevertheless, the effect size of the sleep outcome in this study needs to be interpreted cautiously since some studies that target sleep difficulties specifically have suggested that the clinical efficacy of CBT should reduce the global PSQI score by at least 25% (Wei et al., 2024; Ye et al., 2015).

Our findings support the results from the PACE study, which found that CBT significantly improved sleep problems measured by the Jenkins Sleep Scale (JSS) compared to APT or SMC (White et al., 2011). The JSS was originally developed to assess sleep problems, specifically insomnia symptoms, while the PSQI, as used in our study, is considered a gold standard for self-perceived sleep quality (Fabbri et al., 2021) and is a more general sleep questionnaire covering several sleep disorders (Fabbri et al., 2021). 

The sleep components of CBT in this study included sleep hygiene, education, bed restriction, and stimulus control. Bed restriction and stimulus control, the essential techniques in cognitive behavioural therapy for insomnia (CBT-I), are strongly recommended by the American Academy of Sleep Medicine (AASM) and the European Sleep Research Society (ESRS) (Edinger et al., 2021; Riemann et al., 2023). The study by Gotts et al. (2016) found evidence for the efficacy of CBT-I in CFS/ME patients who complied with the intervention. Additionally, the study by Kallestad et al. (2015) found that Acceptance and Commitment Therapy (ACT) decreased insomnia severity, which predicted improvement in fatigue. The primary goal of treating chronic insomnia is to improve sleep quality and reduce the impact of insomnia (Edinger et al., 2021; Riemann et al., 2023). In this study, we found that sleep quality was improved by including a sleep component in the CBT intervention. 

Fatigue and physical functioning, as well as work and social adjustment, were also improved significantly from baseline. These factors were correlated with sleep quality. Inadequate sleep intensifies fatigue and physical limitations, while increased tiredness and lower physical activity contribute to poorer sleep, forming a self-perpetuating cycle (Kujawski et al., 2021).

### Strengths

Our study had several strengths: 1) The intervention was delivered by specialist cognitive behaviour therapists using an approach that had already been evaluated in an RCT (White et al., 2011). 2) We used a gold standard sleep measure to assess sleep difficulties. In addition, we examined the internal consistency of the PSQI in CFS/ME participants using Cronbach's alpha. The result showed that the PSQI was found to be reliable. The confirmatory analysis also showed a statistically positive regression coefficient, which confirmed the pre-defined theoretical model of the PSQI (Tavakol & Dennick, 2011). 3) This study was longitudinal and followed participants for up to 12 months in a routine clinic.

### Limitations

Some limitations need to be considered. 1) This was a cohort study in a real-world clinical setting, so there was no comparator. Hence, it is impossible to make a causal inference that CBT alone, without the influence of extraneous factors, improves sleep outcomes. To deal with this issue, future studies should include a waiting list control sample within naturalistic settings. 2) The evaluation of sleep was based on the PSQI, which is strongly related to psychological symptom ratings and sleep diary measures; there was no objective measure, such as polysomnography or actigraphy, to confirm or exclude sleep-related disorders. Future studies should thoroughly evaluate sleep-related disorder risk factors, especially obstructive sleep apnoea and narcolepsy, which could contribute to fatigue. Polysomnography is likely to be a helpful investigation for some patients. In addition, the insomnia severity index (ISI) could be used in routine clinical practice, since the insomnia phenotype was reported to be common in CFS/ME patients (Morin et al., 2011). 3) There was a high attrition rate; 48.4% of participants completed the discharge questionnaires. However, Roseborough et al. (2016) estimated that the discontinuation rate of psychotherapy in all clients across populations and settings is between 20 and 57 per cent. 4) Our sample was from one specialist clinic, so it may not represent all CFS/ME patients in other settings. 5) Information about hypnotics and psychotropics use was not included in this study; hence, we could not demonstrate whether the hypnotic use rate changed across time points.

### Implications and Future Research

This was the first study to explore the effectiveness of CBT on sleep outcomes, including sleep quality, sleep latency, sleep duration, and sleep efficiency in a real-world clinical service. The results showed that poor sleep quality was highly prevalent in CFS/ME. Furthermore, CBT leads to positive changes in sleep quality, sleep latency, and sleep efficiency. Interestingly, the effect of CBT on sleep outcomes persisted over time to at least a 12-month follow-up period. This suggests that CBT is an effective intervention with lasting effects. One potential explanation for the long-lasting effect is that patients can continue to apply CBT in managing their symptoms after therapist-aided CBT has ceased.

Future studies should consider control conditions such as a waiting list. It would be beneficial to explore which elements of CBT are determinants of positive sleep outcomes and explore potential mechanisms. Data on hypnotic and psychotropic use, including type, dosage, frequency, and duration, should be evaluated as medication could be a confounder affecting sleep quality. Polysomnography or actigraphy may be used together with subjective questionnaires to evaluate the effect of CBT on sleep parameters more specifically. Finally, strategies to reduce the dropout rate and increase CBT adherence in CFS/ME patients are important.

### Conclusion

This is the first study to explore the effectiveness of CBT on sleep parameters in a cohort of CFS/ME patients seen in a routine clinic. We found a high prevalence of poor sleep quality in our cohort. In addition, CBT significantly and positively affected sleep parameters, including sleep quality, sleep latency, and sleep efficiency, as well as most secondary outcomes.

## Supplementary Materials

For this article, the available supplements include data, materials, code, analysis, and dictionary (see Chinvararak, 2025).



ChinvararakC.
 (2025). Effectiveness of cognitive behavioural therapy on sleep outcome for patients with chronic fatigue syndrome in a routine clinical service
[Data, code, materials]. PsychOpen. https://osf.io/6tuzj


## Data Availability

For this article, data is freely available (see Chinvararak, 2025).

## Appendix

**Table A1:** Consistency of the PSQI Component of Patients With CFS/ME (n = 294)

PSQI Component	Item-total correlation	Cronbach's alpha if item deleted	
Subjective sleep quality	0.540	0.679	Component 1
Sleep latency	0.506	0.684	Component 2
Sleep duration	0.567	0.669	Component 3
Habitual sleep efficiency	0.482	0.690	Component 4
Sleep disturbance	0.514	0.694	Component 5
Use of sleeping medication	0.290	0.749	Component 6
Daytime dysfunction	0.310	0.726	Component 7
Cronbach's alpha		0.731	

**Table A2:** Correlation Between the PSQI Component of Patients With CFS/ME

PSQI Component	1	2	3	4	5	6	7	8
1. Subjective sleep quality	1.000							
								
2. Sleep latency	.338**	1.000						
	< .001							
3. Sleep duration	.469**	.324**	1.000					
	< .001	< .001						
4. Habitual sleep efficiency	.310**	.337**	.599**	1.000				
	< .001	< .001	< .001					
5. Sleep disturbance	.469**	.390**	.355**	.260**	1.000			
	< .001	< .001	< .001	< .001				
6. Use of sleeping medication	.225**	.323**	.147*	.173**	.206**	1.000		
	< .001	< .001	.011	.003	< .001			
7. Daytime dysfunction	.296**	.215**	.250**	.146*	.328**	.079	1.000	
	< .001	< .001	< .001	.012	< .001	.176		
8. Global PSQI Score	.681**	.678**	.716**	.668**	.626**	.547**	.473**	1.000
	< .001	< .001	< .001	< .001	< .001	< .001	< .001	

**p* < .05. ***p* < .01.

**Table A3:** Factor Structure of the PSQI Component

PSQI Component	B	95% CI	SE	*z*	*p*
Subjective sleep quality (C1)	0.667	0.571, 0.764	0.049	13.590	< .001
Sleep latency (C2)	0.555	0.450, 0.660	0.054	10.330	< .001
Sleep duration (C3)	0.545	0.434, 0.655	0.056	9.690	< .001
Habitual sleep efficiency (C4)	0.446	0.330, 0.562	0.059	7.560	< .001
Sleep disturbance (C5)	0.692	0.598, 0.786	0.048	14.480	< .001
Use of sleeping medication (C6)	0.303	0.175, 0.430	0.065	4.660	< .001
Daytime dysfunction (C7)	0.435	0.320, 0.549	0.058	7.450	< .001

*Note*. B = Regression coefficient; SE(B) = Standard error of B.

**Table A4:** Fit Indices of the One-Factor Structure

Fit index	χ*^2^*	*df*	χ*^2^/df*	*p*	CFI	TLI	RMSEA	SRMR
Level of acceptance			< 3.00	> .05	> 0.90	> 0.90	< 0.08	< 0.05
Adjusted model	11.98	11	1.09	.365	0.998	0.996	0.017	0.028

*Note*. χ^2^ = Chi-squared test; *df* = degrees of freedom; CFI = comparative fit index; RMSEA = root mean square error of approximation; SRMR = standardized root mean square residual; NFI = normed fit index; TLI = Tucker-Lewis index.
